# Surgical management of raised ICP in craniosynostosis: experience-based selection of posterior vault expansion techniques

**DOI:** 10.1007/s00381-025-06961-8

**Published:** 2025-10-02

**Authors:** Amparo Saenz, Andrea Allen-Tejerina, Alexandra Didi Leslie-Pyke, Luke Smith, Silvia Schievano, Adikarige Haritha Dulanka Silva, Greg James, David Dunaway, Juling Ong, Owase Jeelani

**Affiliations:** 1 Pediatric Neurosurgery Department, Great Ormond Street Hospital, London, UK; 2 Craniofacial Unit, Great Ormond Street Hospital, London, UK; 3 Face Value Research Group, Great Ormond Street Hospital, London, UK

**Keywords:** Craniosynostosis, Elevated intracranial pressure, Pediatric, Surgical outcomes

## Abstract

**Objective:**

To evaluate outcomes of three posterior vault expansion (PVE) techniques in children with craniosynostosis and confirmed raised intracranial pressure (ICP) and propose a practical framework to guide surgical technique selection based on patient-specific characteristics.

**Methods:**

We retrospectively analyzed 116 pediatric patients who underwent their first PVE for confirmed raised ICP between January 2018 and January 2024 at a tertiary craniofacial center. Patients underwent one of three surgical techniques: static remodeling (PVE-S), spring-assisted posterior vault expansion-classic (SAPVE-C), or spring-assisted posterior vault expansion-vertical vector (SAPVE-VV). Outcomes included functional improvement (fundoscopy, VEPs, symptoms, Chiari I), volumetric change in intracranial volume (ICV), operative time, transfusion rates, complications, and reoperation-free survival. Analyses were stratified by age and syndromic diagnosis.

**Results:**

All techniques significantly improved functional markers, with complete resolution of papilledema and normalized VEPs in over 80% of patients. SAPVE-C and SAPVE-VV achieved greater increases in ICV compared to PVE-S (18.9% and 17.2% vs. 12.7%, respectively). SAPVE-C was fastest (mean 138 min), followed by SAPVE-VV (174.7 min) and PVE-S (214.5 min). Transfusion rates were highest in SAPVE-C (64.1%) and SAPVE-VV (60.5%). Reoperation occurred in 11.2% of patients, with significantly higher risk in those operated before age 1 (*p* = 0.04). Age-stratified outcomes revealed SAPVE-C achieved the highest volumetric gain in < 2 years, while SAPVE-VV was most effective in older patients.

**Conclusions:**

Each PVE technique offers distinct advantages. SAPVE-C is optimal for syndromic infants, SAPVE-VV provides volumetric control in complex cases across ages, and PVE-S remains effective for older, non-syndromic patients. A tailored, age- and diagnosis-based selection strategy is essential for optimizing outcomes in craniosynostosis-related raised ICP.

**Supplementary Information:**

The online version contains supplementary material available at 10.1007/s00381-025-06961-8.

## Introduction

Craniosynostosis, defined as the premature fusion of cranial sutures, is associated with significant complications, including elevated intracranial pressure (ICP), impaired brain development, and structural brain abnormalities. Elevated ICP, when untreated, can lead to optic atrophy and, in severe cases, vision loss [1, 2]. The neurosurgical approach to craniosynostosis often focuses on decompression and skull expansion to prevent or alleviate functional neurological compromise [3, 4].

Posterior vault expansion (PVE) has emerged as a key technique for increasing intracranial volume (ICV) and managing raised ICP, particularly in complex or syndromic cases, as it expands the posterior two-thirds of the skull and delivers a greater increase in intracranial volume compared to other approaches [5]. 

At Great Ormond Street Hospital for Children Foundation Trust (GOSH), three posterior vault techniques have been adopted over the past two decades: traditional static remodeling (PVE-S) [6], spring-assisted posterior vault expansion in the classic configuration (SAPVE-C) [7], and a modified spring-assisted vertical vector technique (SAPVE-VV), developed to optimize multidirectional control in complex skull deformities.

Although prior retrospective studies have evaluated outcomes of various cranial expansion methods, few have focused on decision-making frameworks that incorporate both functional outcomes and patient characteristics such as age, syndromic diagnosis, and cranial morphology [8–10]. More recently, Udayakumaran et al. [11] emphasized the importance of tailoring posterior calvarial vault distraction according to volume, venous anatomy, and vector considerations in syndromic craniosynostosis, underscoring the need for individualized approaches to optimize functional outcomes. While their work highlights the nuanced application of distraction-based posterior vault expansion, our study represents the first comprehensive analysis to include static remodeling and spring-assisted posterior vault expansion techniques. By directly comparing these modalities, we propose a practical, experience-based framework that incorporates both functional outcomes and patient-specific variables into surgical decision-making.

Although prior retrospective studies have evaluated outcomes of various cranial expansion methods, few have focused on decision-making frameworks that incorporate both functional outcomes and patient characteristics such as age, syndromic diagnosis, and cranial morphology. Optimizing surgical outcomes requires selecting the most appropriate technique, considering patient-specific factors such as underlying pathology, age, and cranial morphology.

This study presents our institutional experience using three PVE techniques in a cohort of patients with craniosynostosis and confirmed raised ICP. We analyze functional outcomes, complication rates, intracranial volume changes, and reoperations, and propose a practical selection framework to guide individualized surgical decision-making. 

## Methodology

### Study design and setting

This retrospective cohort study was conducted at Great Ormond Street Hospital (GOSH), a tertiary pediatric neurosurgical and craniofacial center in the UK. We included all patients with a diagnosis of craniosynostosis who underwent their first posterior vault expansion (PVE) procedure for raised ICP between January 2018 and January 2024, with a minimum of 12 months postoperative follow-up.

Inclusion was limited to patients who underwent surgery for functional indications, defined as suspected or confirmed elevated ICP. Exclusion criteria included patients who underwent PVE exclusively for aesthetic correction or morphological remodeling, as well as patients with prior PVE procedures or incomplete postoperative data.

Raised ICP was diagnosed based on institutional criteria, which included one or more of the following: abnormal findings on invasive ICP monitoring, the presence of papilledema on fundoscopy, altered visual evoked potentials (VEPs), or a Chiari I malformation—defined as tonsillar descent greater than 7 mm below the McRae line—associated with syringomyelia and characteristic symptoms such as occipital headache and sleep-related disturbances. In cases where only indirect radiological signs were present (e.g., copper-beaten skull, mildly enlarged ventricles, or isolated Chiari I malformation without syrinx) or where the patient presented solely with symptoms, ICP monitoring was performed to confirm the presence of intracranial hypertension before offering surgery. At GOSH, posterior vault expansion is not performed prophylactically; surgical intervention is reserved for cases with objective evidence of raised ICP.

Data collected included demographic variables (age at surgery, sex), diagnostic classifications (type of craniosynostosis and syndromic diagnosis), and surgical technique used. The study was approved by the institutional research board at GOSH, and all data were handled in compliance with relevant data protection regulations.

## Operative Techniques

### Posterior vault expansion static (PVE-S)

The PVE-S surgeries included in this study are a modified version of a previously published technique [6, 8], where the expansion is limited to the posterior two-thirds of the cranial vault (Online Resource 1a-b).

The patient is placed in a prone position with a bicoronal incision. The skin flap is reflected, and the pericranium is dissected. The static technique slightly varies depending on the underlying skull morphology, but it always involves two lateral panels and a strip of bone behind the coronal strip (3–4 cm) that is rotated and repositioned to recontour the occiput [12]. A posterior strip over the torcula is removed from the parietal pieces for better control of the veins during the osteotomies. It is then replaced to broaden the biparietal region. The construct is secured with plates and screws. The pericranium is closed, followed by the scalp with absorbable sutures.

### Spring-assisted posterior vault expansion classic technique (SAPVE-C)

SAPVE-C follows the methodology previously described by the GOSH surgical team [6–8] (Online Resource 2).

The patient is placed in a prone position with a bicoronal incision and pericranial flaps for coverage. A bucket-handle osteotomy is performed, extending across the transverse sinuses toward the foramen magnum. Burr holes are strategically placed to minimize venous sinus injury. Springs are positioned across the osteotomies to provide expansion force. The number and size of springs are chosen based on the required expansion. The pericranium is closed over the springs, followed by scalp closure with absorbable sutures (Video, Supplemental Digital Content 1).

### Spring-assisted posterior vault expansion vertical vector technique (SAPVE-VV)

SPVE-VV was developed to enhance adaptability in cranial expansion as it modifies the expansion vector towards an upward and backward rotation and allows simultaneous static lengthening and widening (Online Resource 3). 

The patient is placed in a prone position with a bicoronal incision. The pericranium is raised in flaps, which provide soft tissue cover over the springs and bone gaps.

The osteotomies are designed to achieve dynamic expansion in a vertical vector, with options to adjust expansion in the anteroposterior and horizontal planes, depending on the patient’s needs. The technique allows the skull to expand upwards rather than just posteriorly. The parietal bones are entirely removed along with the occipital bone at the level or below the torcula. Static expansion can be achieved with plates and screws to allow widening or lengthening of the construct. In scaphocephaly, the technique also allows shortening of the construct if necessary. Springs are placed laterally to gradually expand the skull over time. The pericranium is closed over the springs, and the scalp is closed with absorbable sutures (Video, Supplemental Digital Content 2).

### Outcomes of interest

The study’s primary outcomes were functional improvement, intracranial volume (ICV) change, complication rate, transfusion requirement, operative time, length of hospital stay, and reoperation rate. Functional improvement was assessed based on the resolution or improvement of raised ICP markers, including papilledema, altered visual evoked potentials (VEPs), tonsillar descent, syrinx, and documented symptoms such as headache, irritability, vomiting, full fontanelle, or sleep disturbance. Ophthalmologic assessments and VEP testing were performed pre- and postoperatively as part of the standard multidisciplinary care pathway. Chiari I malformation was defined as cerebellar tonsillar herniation of 7 mm or more below the foramen magnum, and syringomyelia was evaluated on pre- and postoperative magnetic resonance imaging. Patients with radiologically confirmed Chiari I were considered improved only if there was both radiological regression and clinical symptom resolution.

ICV was measured using volumetric analysis of pre- and postoperative computed tomography (CT) scans. Only CT scans performed within 6 months preoperatively and up to 12 months postoperatively were considered for volumetric comparison. Complications included wound infections, spring exposure, cerebrospinal fluid (CSF) leaks, and any postoperative event requiring surgical intervention. Transfusion requirement was recorded if blood products were administered intraoperatively or within 24 h after surgery. Transfusion was indicated for hemoglobin levels below 70 g/dL, or between 70 and 80 g/dL if symptomatic. Operative time was defined from skin incision to final suture placement. Length of hospital stay was recorded as the total number of days from the date of surgery to discharge.

Reoperation was defined as any subsequent intracranial procedure indicated for persistent or recurrent signs of raised ICP, such as positive ICP monitoring, new-onset papilledema, symptomatic Chiari I malformation, or deterioration in VEPs. Planned secondary procedures, such as spring removal or aesthetic corrections unrelated to functional deterioration, were not classified as reoperations for this analysis.

### Statistical analysis

Continuous variables were reported as means with standard deviations (SD) and categorical variables as absolute frequencies and percentages. Between-group comparisons for continuous variables were conducted using one-way analysis of variance (ANOVA), with Bonferroni correction for multiple comparisons when applicable. Categorical variables were compared using the chi-squared test or Fisher’s exact test, depending on sample size distribution. Pre- and postoperative changes in intracranial volume (ICV) were evaluated using paired *t*-tests.

Functional outcomes, including papilledema, altered VEPs, Chiari I malformation, and symptoms suggestive of raised ICP, were analyzed using McNemar’s test to assess changes between baseline and follow-up assessments. Reoperation-free survival was estimated using Kaplan–Meier survival analysis, and differences between surgical technique groups were assessed using the log-rank test. Cox proportional hazards regression was used to evaluate the association between surgical technique and risk of reoperation, adjusting for potential confounders, including syndromic diagnosis and age under 2 years. Incidence rates of reoperation were expressed as events per 1000 patient-years.

To explore the relationship between age at surgery and absolute ICV gain, linear regression was performed separately within each surgical technique group. An interaction model was also constructed to assess whether the association between age and ICV gain varied by surgical technique. All regression models reported coefficients with 95% confidence intervals and *p*-values.

Statistical significance was defined as a *p*-value < 0.05. All analyses were performed using Stata IC/16.1 (StataCorp, College Station, TX, USA).

## Results

A total of 116 patients met the inclusion criteria for this study, all of whom underwent posterior vault expansion for confirmed raised intracranial pressure. Of these, 34 underwent PVE-S, 39 underwent SAPVE-C, and 43 underwent SAPVE-VV.

The mean age at surgery for the entire cohort was 46.3 months (SD 36.8). The distribution of patients by age group and syndromic status varied across surgical techniques. SAPVE-C was most often performed in younger patients, particularly those under 2 years of age (71.8%), and in syndromic cases (87.2%). In contrast, PVE-S was primarily used in older, non-syndromic patients, while SAPVE-VV was applied across a broader age range and included a mix of syndromic (48.8%) and non-syndromic cases. The most common syndromic diagnoses were Crouzon syndrome (14.7%), Apert syndrome (9.5%), and Pfeiffer syndrome (9.5%). Most cases involved multisuture or pansynostosis, although single-suture patients with functional indications were also included, accounting for 16.4% of the cohort. Baseline characteristics by surgical technique are summarized in Table 1.

**Table 1:** Baseline characteristics of the study population stratified by posterior vault expansion technique

Characteristic	PVE-S (*n* = 34)	SAPVE-C (*n* = 39)	SAPVE-VV (*n* = 43)	Total (*n* = 116)
Age at surgery, mean (SD), mo	74.7 (33.8)	20.3 (19.8)	47.5 (34.4)	46.3 (36.8)
Age < 2 years, *n* (%)	2 (5.9%)	28 (71.8%)	10 (23.3%)	40 (34.5%)
Age 2–4 years, *n* (%)	4 (11.8%)	7 (17.9%)	16 (37.2%)	27 (23.3%)
Age > 4 years, *n* (%)	28 (82.4%)	4 (10.3%)	17 (39.5%)	49 (42.2%)
Syndromic diagnosis, *n* (%)	17 (50.0%)	34 (87.2%)	21 (48.8%)	72 (62.1%)
Apert syndrome	2 (5.9%)	6 (15.4%)	3 (7.0%)	11 (9.5%)
Crouzon syndrome	3 (8.8%)	9 (23.1%)	5 (11.6%)	17 (14.7%)
Pfeiffer syndrome	3 (8.8%)	6 (15.4%)	2 (4.7%)	11 (9.5%)
Craniosynostosis type				
Single-suture	8 (23.5%)	0 (0.0%)	11 (25.6%)	19 (16.4%)
Bicoronal	20 (58.8%)	24 (61.5%)	21 (48.8%)	65 (56.0%)
Multisuture	26 (76.5%)	38 (97.4%)	32 (74.4%)	96 (82.8%)
Pansynostosis	25 (73.5%)	25 (64.1%)	22 (51.2%)	72 (62.1%)
Ventriculoperitoneal shunt before PVE surgery, *n* (%)	2 (5.9%)	5 (12.8%)	3 (7.0%)	10 (8.6%)

### Functional outcomes

**Fig. 1:**
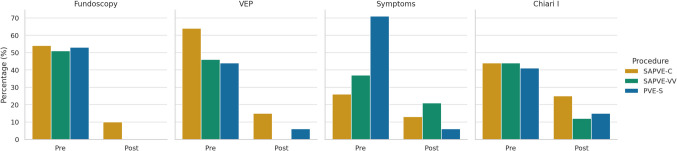
Bar graph showing preoperative and postoperative presence of papilledema, visual evoked potential alterations, symptoms of raised intracranial pressure, and Chiari I malformation, stratified by surgical technique (SAPVE-C, SAPVE-VV, PVE-S). Percentage of patients with each finding is plotted on the y-axis

Preoperative assessment identified functional abnormalities in a substantial proportion of the cohort. Figure 1 summarizes the results of the functional outcomes.

Papilledema was observed in over half of the patients across all three surgical groups, 52.9% in PVE-S, 53.8% in SAPVE-C, and 51.2% in SAPVE-VV. Following posterior vault expansion, complete resolution was noted in all cases treated with PVE-S and SAPVE-VV, and in 81.0% of those treated with SAPVE-C. These changes were statistically significant in all groups (*p* < 0.001 for PVE-S and SAPVE-VV, *p* < 0.001 for SAPVE-C).

Altered visual evoked potentials (VEPs) were present preoperatively in 44.1% of PVE-S, 64.1% of SAPVE-C, and 46.5% of SAPVE-VV patients. Postoperative normalization occurred in 86.7%, 84.0%, and 100% of affected patients in each respective group, all reaching statistical significance (*p* < 0.001, *p* < 0.001, and *p* < 0.001).

Symptoms consistent with raised intracranial pressure, including headaches, irritability, nausea, vomiting, sleep disturbances, and behavioral changes, were present in 70.6% of PVE-S, 25.6% of SAPVE-C, and 37.2% of SAPVE-VV patients at baseline. Follow-up, symptom resolution, or marked improvement was observed in 94.1% of PVE-S patients (*p* < 0.001). Improvements were also seen in SAPVE-C (87.2%) and SAPVE-VV (79.1%) groups, though these did not reach statistical significance (*p* = 0.125 and *p* = 0.065, respectively).

Chiari I malformation was radiologically identified in 42.4% of PVE-S, 44.7% of SAPVE-C, and 48.7% of SAPVE-VV cases. Postoperative imaging showed improvement in 78.6%, 94.1%, and 84.4% of affected patients, respectively. Statistical significance was achieved in SAPVE-C (*p* = 0.022) and SAPVE-VV (*p* = 0.002), while the change in PVE-S approached significance (*p* = 0.062).

Syringomyelia associated with Chiari I malformation was present preoperatively in four patients from each surgical group. Postoperative follow-up demonstrated radiological improvement of the syrinx in all affected cases.

### Intracranial volume increase

Volumetric data were available for 82 patients, including 26 who underwent PVE-S, 26 SAPVE-C, and 30 SAPVE-VV. All patients had both preoperative and postoperative three-dimensional imaging suitable for volumetric analysis within 12 months of surgery.

For the entire cohort, the mean preoperative intracranial volume was 1164.7 cm^3^ (SD 281.2), and the mean postoperative volume was 1412.7 cm^3^ (SD 253.1), corresponding to a mean absolute increase of 224.7 cm^3^ (SD 162.6) and a relative increase of 16.4% (SD 11.4). The mean preoperative ICV was 1324.3 cm^3^ (SD 210.3) in the PVE-S group, 1026.1 cm^3^ (SD 308.8) in the SAPVE-C group, and 1155.7 cm^3^ (SD 272.6) in the SAPVE-VV group. The corresponding mean postoperative volumes were 1519.7 cm^3^ (SD 183.4), 1264.4 cm^3^ (SD 324.1), and 1439.9 cm^3^ (SD 194.2), respectively. Mean absolute volume increases were 195.4 cm^3^ (SD 170.4) for PVE-S, 238.3 cm^3^ (SD 157.9) for SAPVE-C, and 236.2 cm^3^ (SD 155.2) for SAPVE-VV. The relative increase in volume was 12.7% (SD 10.4) for PVE-S, 18.9% (SD 11.2) for SAPVE-C, and 17.2% (SD 11.8) for SAPVE-VV. Paired *t*-tests confirmed that the increase in intracranial volume from preoperative to postoperative imaging was statistically significant in all three groups (PVE-S: *p* < 0.001; SAPVE-C: *p* < 0.001; SAPVE-VV: *p* < 0.001).

**Fig. 2:**
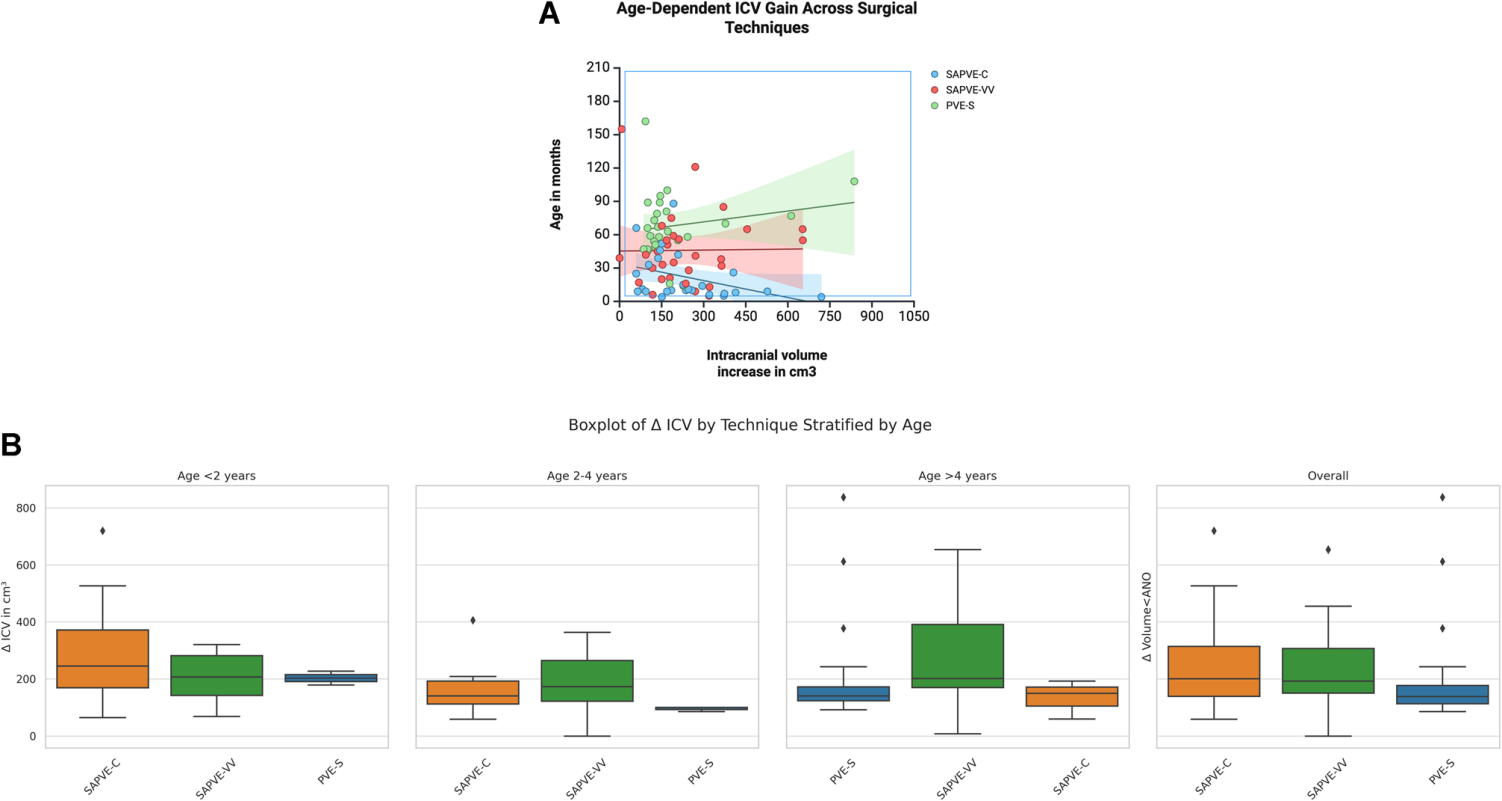
**a** Boxplot of the differential intracranial volume (ICV) in cm^3^, stratified by age group (< 2 years, 2–4 years, > 4 years) and surgical technique (SAPVE-C, SAPVE-VV, PVE-S). ΔICV represents postoperative minus preoperative volume. **b** Age-dependent absolute intracranial volume (ICV) gain (in cm.^3^) across surgical techniques. Each dot represents an individual patient, color-coded by procedure: SAPVE-C (blue), SAPVE-VV (red), and PVE-S (green). Linear regression lines with 95% confidence intervals (shaded areas) are plotted for each group. The y-axis indicates age at surgery in months, and the x-axis shows the absolute postoperative ICV increase. While no statistically significant correlation was found for SAPVE-C or PVE-S, a significant inverse relationship between age and ICV gain was observed in the SAPVE-VV group (*p* = 0.026)

When stratified by age group, patients younger than 2 years exhibited the highest mean percentage increase in intracranial volume. Among patients who underwent SAPVE-C, those under 2 years had a mean ICV increase of 22.9% (SD 10.7; *n* = 17), while those aged 2–4 years had a mean increase of 13.2% (SD 9.1; *n* = 6). In the SAPVE-VV group, the mean ICV increase was 21.2% (SD 13.3; *n* = 8) for patients under 2 years, 14.4% (SD 7.2; *n* = 12) for those aged 2–4 years, and 16.4% (SD 11.7; *n* = 10) for those over 4 years. In the PVE-S group, the mean increase was 15.0% (SD 1.4; *n* = 2) for patients under 2 years, 6.3% (SD 0.6; *n* = 3) for those aged 2–4 years, and 13.5% (SD 11.2; *n* = 21) for patients older than 4 years. These findings are summarized in Fig. 2a, which shows the distribution of relative ICV increase in cm^3^ by surgical technique across age categories.

Linear regression was used to further explore the relationship between age at surgery (in months) and absolute ICV increase within each surgical technique group. In the SAPVE-C cohort, age was significantly associated with reduced ICV gain (β = −3.14, 95% CI −5.88 to −0.40, *p* = 0.026), indicating that younger patients experienced larger volume expansions. No significant association was found for SAPVE-VV (β = −0.35, *p* = 0.823) or PVE-S (*β *= 1.08, *p* = 0.360), although the positive trend in the PVE-S group suggested a possible inclination for greater volume gains in older patients. An interaction model including all techniques did not reach statistical significance (*p* = 0.30), but showed a trend toward different age–volume relationships across techniques. Figure 2b shows these associations with regression lines and 95% confidence intervals plotted by technique.

### Complication rate, spring exposure, and infection

Among the 116 patients included in the study, 23 (19.8%) experienced at least one postoperative complication. Of these, 16 (13.8%) were classified as Oxford Type 1, indicating minor events that did not impact patient outcomes or require intervention. The remaining seven complications (6.0%) were classified as Oxford Type 3, as they required surgical reintervention. No Oxford Type 2, 4, or 5 complications were recorded.

Oxford Type 3 complications occurred in 1 of 34 patients (2.9%) in the PVE-S group, 2 of 39 (5.1%) in the SAPVE-C group, and 4 of 43 (9.3%) in the SAPVE-VV group. In the PVE-S group, the complication involved a surgical drain that had partially dislodged and could not be removed on the ward, requiring removal under general anesthesia. The two SAPVE-C complications were spring extrusions with associated infections, both requiring operative washout and spring removal. Among the SAPVE-VV cases, three patients experienced spring extrusion with concomitant infection requiring early removal and washout, and one patient developed atlantoaxial rotatory fixation necessitating manual correction under general anesthesia.

### Transfusion requirement

**Fig. 3:**
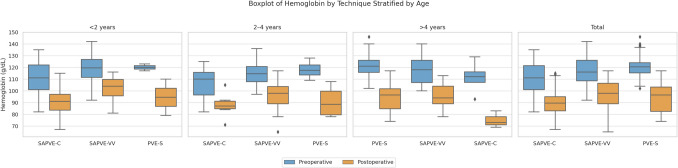
Boxplot of preoperative and postoperative hemoglobin levels (g/dL), stratified by surgical technique (SAPVE-C, SAPVE-VV, PVE-S) and age group (< 2 years, 2–4 years, > 4 years)

Preoperative and postoperative hemoglobin levels were available for all 116 patients. The mean preoperative hemoglobin concentration was 121.9 g/L (SD 10.3) in the PVE-S group, 110.8 g/L (SD 13.3) in the SAPVE-C group, and 117.2 g/L (SD 13.0) in the SAPVE-VV group. Postoperatively, the mean hemoglobin concentrations decreased to 94.2 g/L (SD 11.8), 88.8 g/L (SD 13.1), and 97.1 g/L (SD 12.3), respectively. Figure 3 shows the distribution of the hemoglobin levels before and after surgery by technique across age ranges.

Transfusion rates varied by surgical technique. In the PVE-S group, 12 of 34 patients (35.3%) received a transfusion, compared with 25 of 39 (64.1%) in the SAPVE-C group and 26 of 43 (60.5%) in the SAPVE-VV group.

Logistic regression analysis was performed to assess potential predictors of transfusion. Younger age (defined as ≤ 1 year) was significantly associated with increased odds of receiving a transfusion (OR 2.8, *p* = 0.043), whereas syndromic status was not significantly associated with transfusion risk (*p* = 0.924).

### Duration of the surgery and length of stay

The duration of surgery was recorded for 94 patients. The mean operative time was 214.5 min (SD 57.9) in the PVE-S group (*n* = 32), 138.1 min (SD 55.4) in the SAPVE-C group (*n* = 22), and 174.7 min (SD 43.9) in the SAPVE-VV group (*n* = 40). SAPVE-C procedures were generally shorter, while PVE-S had the longest operative times overall.

**Fig. 4:**
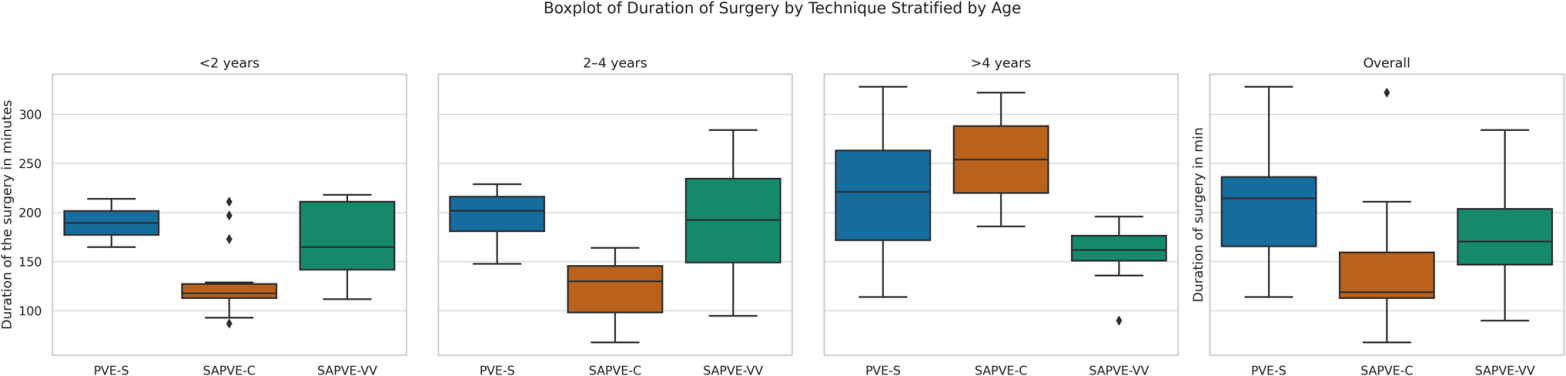
Boxplot of duration of surgery in minutes, stratified by age group (< 2 years, 2–4 years, > 4 years) and surgical technique (SAPVE-C, SAPVE-VV, PVE-S)

To further explore the relationship between surgical duration and patient age, operative time was stratified by age groups (< 2 years, 2–4 years, and > 4 years). Figure 4 shows the distribution of surgical duration by technique across these age ranges.

Length of stay was available for the entire cohort. The median hospital stay was 3 days across all groups. The mean length of stay was 3.6 days (SD 1.2) in the PVE-S group (*n* = 34), 4.0 days (SD 1.7) in the SAPVE-C group (*n* = 39), and 3.5 days (SD 1.2) in the SAPVE-VV group (*n* = 43).

### Reoperation rate

A total of 13 out of 116 patients (11.2%) required a second cranial vault expansion procedure due to signs of persistent or recurrent raised intracranial pressure during follow-up. Reoperations were more frequent in the SAPVE-C group (9 of 39 patients, 23.1%), followed by PVE-S (3 of 34, 8.8%) and SAPVE-VV (1 of 43, 2.3%). Table 2 summarizes the clinical and surgical characteristics of these patients.

**Table 2:** Patients’ clinical and surgical characteristics

Patient	Syndromic diagnosis	Suture involved	Associated findings	Primary indication for surgery	Age at surgery (months)	Initial surgical technique	Reason for reoperation	Interval to reoperation (months)	Secondary procedure	Reoperation outcome
1	Non-syndromic	Pansynostosis	VP Shunt	Positive ICP monitoring	77	PVE-S	Symptoms	29	ICP Monitoring Only	No raised ICP
2	Noonan Syndrome	Sagittal + bicoronal	-	Abnormal VEP	98	PVE-S	Altered VEP/Symptoms	13	FOA	Resolved
3	FGFR2 (Pfeiffer)	Sagittal + bicoronal	-	Abnormal VEP	14	PVE-S	Altered VEP	8	SAPVE-C	Resolved
4	Bartter Syndrome type 4	Pansynostosis	Chiari I	Papilledema/Abnormal VEP	11	SAPVE-C	Altered VEP	8	Positive ICP monitoring/FOA	Resolved
5	FGFR2 (Apert)	Bicoronal synostosis	-	Abnormal VEP	4	SAPVE-C	Altered VEP	21	SAPVE-VV	Resolved
6	FGFR2 (Apert)	Bicoronal synostosis	-	Abnormal VEP	5	SAPVE-C	Altered VEP/Papilledema	28	SAPVE-VV	Resolved
7	FGFR2 (Apert)	Bicoronal + right lambdoid	-	Papilledema	8	SAPVE-C	Altered VEP	28	SAPVE-VV	Resolved
8	FGFR2 (Crouzon)	Bicoronal synostosis	VP shunt/Chiari I	Papilledema	9	SAPVE-C	Symptoms/Chiari I/Syrinx	27	Positive ICP monitoring/SAPVE-VV	Resolved
9	FGFR2 (Crouzon)	Left coronal + bilambdoid	Chiari I/Syrinx	Symptoms	10	SAPVE-C + FMD	Altered VEP	17	Positive ICP monitoring/FOA	Resolved
10	Duchenne muscular dystrophy	Bicoronal + Left Lambdoid	VP Shunt	Papilledema	11	SAPVE-C	Papilledema	54	FOA	Resolved
11	TWIST1 (Saethre Chotzen)	Sagittal + bicoronal	-	Papilledema	14	SAPVE-C	Papilledema	39	Positive ICP monitoring/PVE-s	Resolved
12	FGFR2 (Crouzon)	Pansynostosis	VP Shunt/Chiari I	Papilledema/Abnormal VEP	66	SAPVE-C + FMD	Altered VEP	30	FOA	Resolved
13	Non-syndromic	Sagittal synostosis	-	Positive ICP monitoring	43	SAPVE-VV	Persistent symptoms	19	ICP monitoring only	No raised ICP

**Fig. 5:**
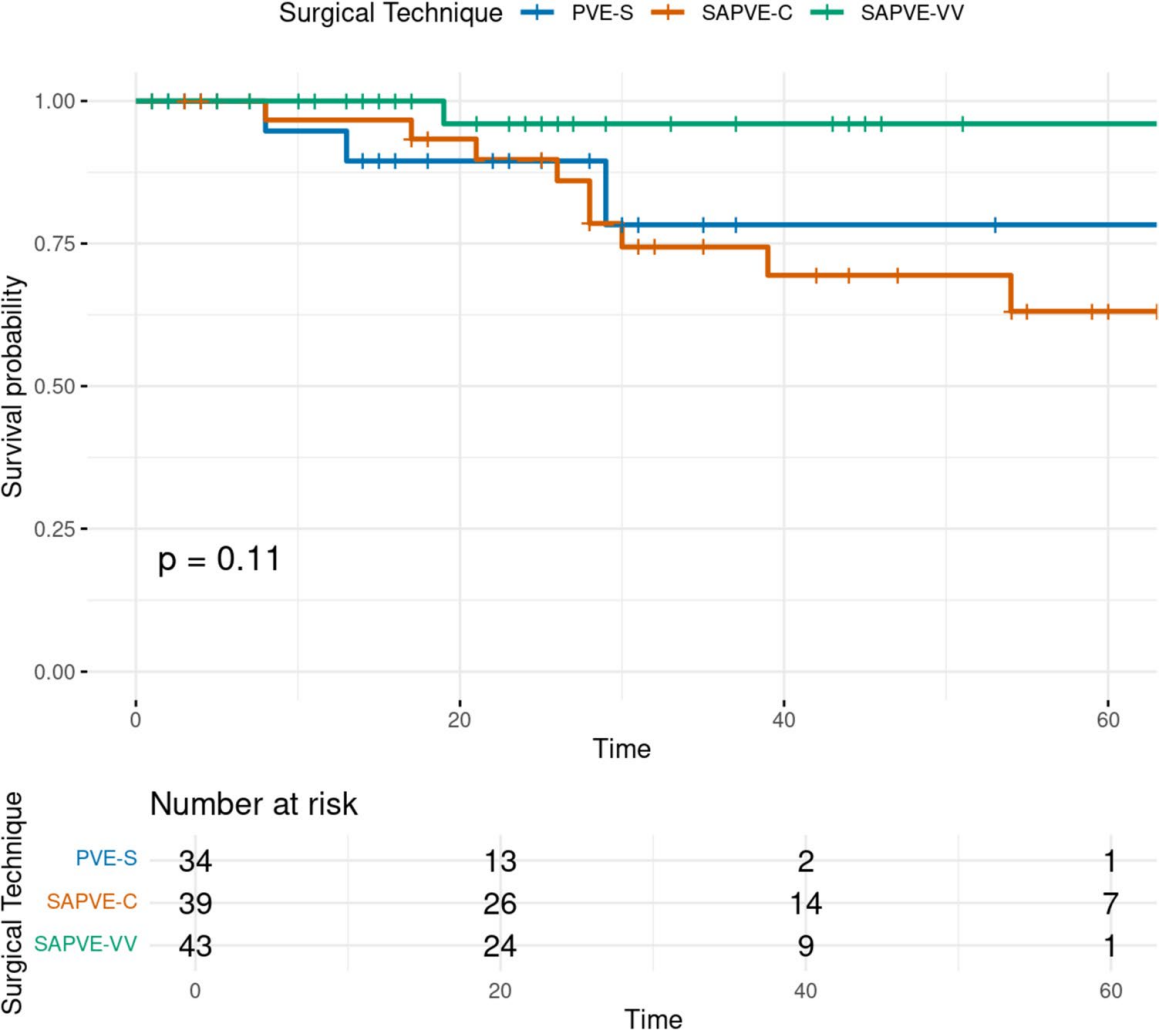
Kaplan–Meier survival curve showing reoperation-free survival by surgical technique. Reoperation due to raised ICP is the event of interest. The survival probability is plotted over time in months. No statistically significant difference was observed (*p* = 0.11)

The total time at risk was 2847 patient-months. The incidence rate of reoperation was 7.21 per 1000 patient-months in the SAPVE-C group, 5.37 per 1000 in the PVE-S group, and 0.96 per 1000 in the SAPVE-VV group. Kaplan–Meier survival curves demonstrated reduced reoperation-free survival in the SAPVE-C group compared to PVE-S and SAPVE-VV (Fig. 5).

**Fig. 6:**
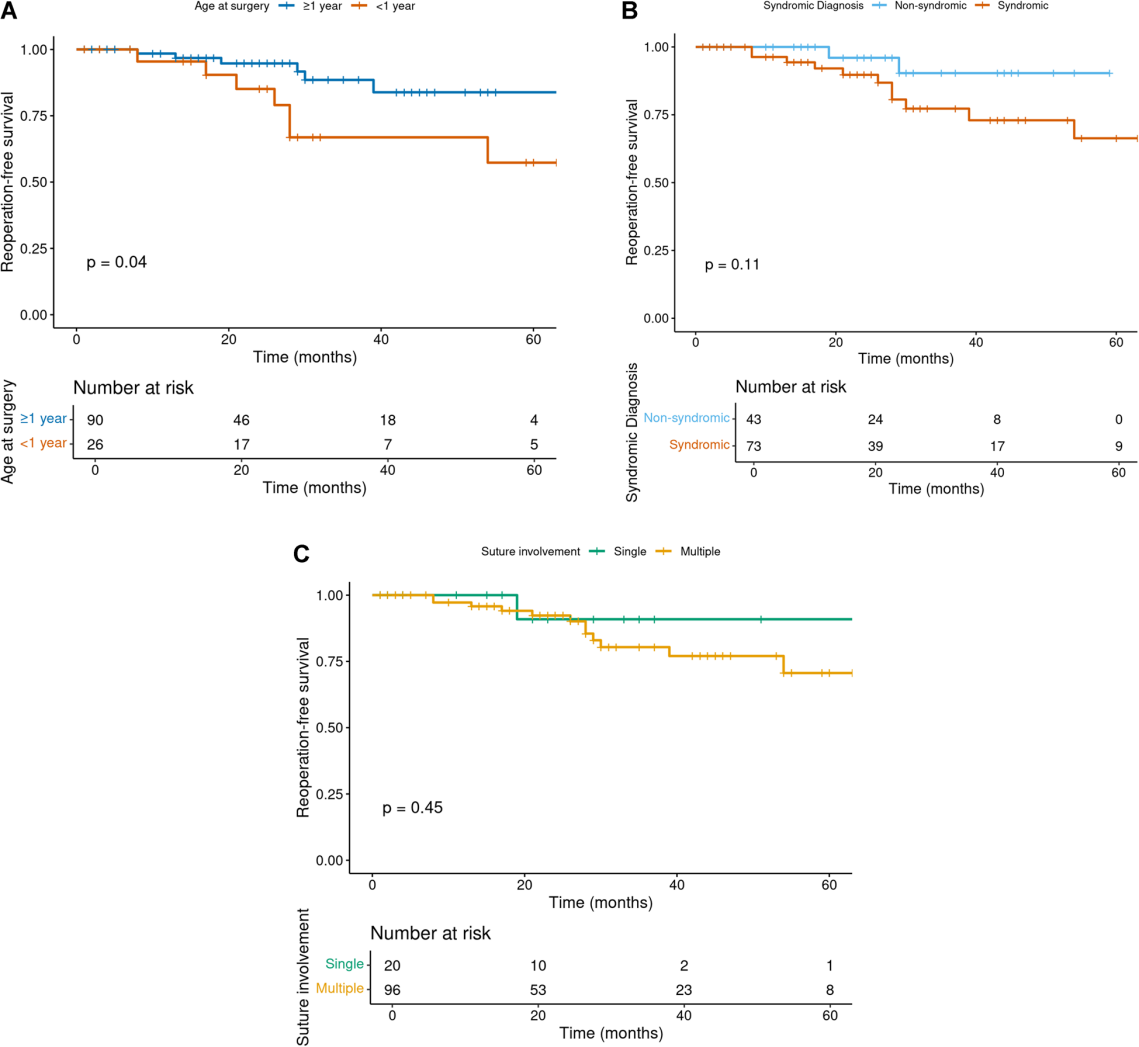
**a** Kaplan–Meier survival curve showing reoperation-free survival stratified by age at surgery (< 1 year vs ≥ 1 year). Patients operated on before the age of 1 year had significantly reduced survival probability (log-rank *p* = 0.04). **b** Kaplan–Meier survival curve showing reoperation-free survival stratified by syndromic diagnosis (syndromic vs non-syndromic craniosynostosis). Although syndromic patients showed lower survival, the difference was not statistically significant (log-rank *p* = 0.11). **c** Kaplan–Meier survival curve showing reoperation-free survival stratified by suture involvement (single vs multiple). Although multisuture craniosynostosis patients showed lower survival, the difference was not statistically significant (log-rank *p* = 0.45)

To explore broader clinical predictors of reoperation independent of surgical technique, Kaplan–Meier survival analysis was stratified by suture involvement, syndromic diagnosis, and age at surgery. Patients who underwent surgery before the age of 1 year had a significantly higher risk of reoperation compared to those operated on at or after 1 year of age (log-rank *p* = 0.040; Fig. 6a). Although patients with syndromic diagnoses showed a trend toward earlier reoperation compared to non-syndromic patients, this difference did not reach statistical significance (log-rank *p* = 0.113; Fig. 6b). Similarly, reoperation-free survival was lower in patients with multiple suture involvement compared to those with a single fused suture, but the difference was not statistically significant (log-rank *p* = 0.454; Fig. 6c).

## Discussion

As surgeons, we constantly refine our techniques to improve outcomes. Around 20 years ago, PVE-S was the only technique offered. In 2008, SAPVE-C was introduced, incorporating gradual expansion devices that added a dynamic element to the expansion process. The benefit of the SAPVE-C over the PVE-S was limited surgical dissection without the need to strip the scalp and the dura completely from the calvarium, which is beneficial for younger children. Larger intracranial volume increases were achieved without compromising scalp integrity. Our initial results with SAPVE-C, published after 200 cases, demonstrated its safety and efficacy, allowing the skin to close with acceptable tension levels while increasing the ICV up to 284.1 ± 171.6 cm^3^ [10].

Although SAPVE-C has proven effective, we identified a subset of patients for whom a more tailored cranial shape control was desirable while maintaining the benefits of spring-assisted expansion. This led to the development of the SAPVE-VV technique, which allows width, length, and vertical height adjustments depending on each patient’s cranial morphology. Although it achieves superior ICV increases compared to PVE-S, the main drawback compared to SAPVE-C is the need for a full craniotomy and bone removal before reconstruction.

When analyzing functional improvements and reoperation rates for secondary raised ICP, we observed a higher reoperation rate in the SAPVE-C group. However, this was largely influenced by the fact that 87% of patients in this group were syndromic, and 71% were younger than 2 years—factors known to increase the risk of secondary surgery [13–15]. Notably, VEPs were the sole indicator of raised ICP in seven cases, proving the importance of this specific neuro-ophthalmological exam in the follow-up of patients with craniosynostosis [16, 17].

Regarding the increase in ICV, spring techniques demonstrated better outcomes than the static technique [10]. SAPVE-C showed the highest overall ICV increase, particularly in patients under 2 years of age, where its impact was most pronounced. This age-dependent effect was further supported by our regression analysis, which showed a significant inverse correlation between age at surgery and ICV gain for SAPVE-C. These findings suggest that SAPVE-C is especially suited for younger patients, in whom early intervention can yield substantial cranial expansion while maintaining minimal invasiveness. SAPVE-VV, on the other hand, showed no significant correlation between age and ICV gain, with a relatively flat regression slope. This suggests that its capacity for expansion is largely age-independent, providing consistent volumetric gains even in older patients. In the 2–4-year age group, SAPVE-C and SAPVE-VV demonstrated comparable ICV increases, while in children older than 4 years, SAPVE-VV outperformed both SAPVE-C and PVE-S. This challenges previous assumptions that spring techniques lose effectiveness as the skull matures and thickens. Instead, our findings indicate that SAPVE-VV remains a powerful option across age ranges and may offer particular advantages in patients requiring substantial expansion beyond early childhood. Although PVE-S showed lower overall ICV gains, our scatterplot analysis revealed a mild trend toward increased volume with advancing age, which, while not statistically significant, may suggest that this technique benefits from more mature bone architecture.

The complication rates for all three techniques were low, with spring techniques offering some advantages over other distraction methods like posterior vault distraction osteogenesis (PVDO). Unlike semi-buried distractors used in PVDO, springs in cranial expansion remain beneath the scalp as part of a “sealed system,” which may explain the reduced infection risk. PVDO has a reported infection rate of 27% and a CSF leak rate of 19% [18], while spring techniques in our cohort showed a 6–8% infection rate and a 0.6% CSF leak rate. Notably, no device malfunctions were observed in this study, compared to 7% with other distraction devices [7].

The higher transfusion rates for spring techniques (65% for SAPVE-C and 60.8% for SAPVE-VV) compared to PVE-S (43.7%) are likely due to patient demographics rather than surgical complexity. Spring techniques are more commonly performed in younger patients, especially those under 2 years, who are more vulnerable to blood loss due to their smaller blood volume. Additionally, many younger patients in the SAPVE-C group were syndromic, a population prone to increased bleeding risks [19, 20]. Syndromic craniosynostosis patients often present with venous hypertension [21], which can worsen bleeding, and typically undergo surgery at an earlier age due to the higher risk of raised ICP. While SAPVE-C is less disruptive to cranial anatomy, patient age and syndromic status are likely the main contributors to the higher transfusion rates, rather than the technique itself [22, 23]. Furthermore, hemoglobin dynamics in our cohort suggest that patients undergoing SAPVE-C started with lower preoperative hemoglobin levels compared to those treated with SAPVE-VV or PVE-S. This trend was especially evident in patients under 2 years of age, reinforcing the influence of younger age and syndromic diagnosis on baseline hematologic status. These lower baseline hemoglobin values mean that even moderate intraoperative blood loss may bring patients closer to transfusion thresholds. Thus, the higher transfusion rates observed in the SAPVE-C group likely reflect not only intraoperative losses but also a lower margin for physiological compensation.

The differences in operative time were notable, with SAPVE-C being the quickest at 127.5 min, followed by SAPVE-VV at 175.3 min and PVE-S at 215.7 min. SAPVE-C’s shorter duration is due to its minimally invasive nature, involving less dissection and cranial anatomy disruption. Although SAPVE-VV requires complete detachment of the bone from the dura, like PVE-S, it is still faster due to a simpler reconstruction phase. Unlike PVE-S, which requires meticulous reassembly of the cranial construct, SAPVE-VV only requires securing the upper part of the construct with plates and adding two springs for expansion. When stratified by age, the data further highlight how surgical efficiency varies across developmental stages. SAPVE-C maintained the shortest and most consistent operative time in children under 4 years, making it a particularly suitable option for younger patients where minimizing anesthesia time is critical. However, in patients older than 4 years, the duration and variability of SAPVE-C increased considerably, possibly reflecting greater anatomical complexity or the challenges of performing limited-access techniques in larger skulls. Conversely, SAPVE-VV showed greater operative time variability in younger patients but emerged as the most time-efficient and consistent approach in older children. PVE-S, while consistently the longest procedure across all age groups, may still be preferable in cases requiring more extensive reshaping.

Hospital stay length was consistent across all techniques, with a mean of 3.5 days for PVE-S and SAPVE-C, and 3.4 days for SAPVE-VV, showing no significant differences. This consistency in recovery time, despite variations in surgical complexity, transfusion rates, and duration, underscores the safety of these procedures.

At GOSH, patient care is guided by a multidisciplinary approach, with each case being thoroughly discussed in our weekly meetings to determine the most appropriate surgical technique based on individual patient needs. While the recommendations outlined here are not rigid, they are based on our institutions’ experience performing these techniques.

For patients < 2, especially those with syndromic craniosynostosis, we prefer SAPVE-C since it is the quickest and simplest technique, provides significant postoperative ICV expansion, and is the least disruptive, leaving the dura attached to the bone for seamless expansion. These benefits are reinforced by our regression analysis, which showed that SAPVE-C achieves greater ICV gains when performed at a younger age, making it especially suitable in early infancy. SAPVE-VV can be considered in this age group in certain indications, such as in scaphocephaly, where the SAPVE-C could exacerbate the deformity.

The choice for patients aged 2–4 years depends on syndromic status, as syndromic patients carry a higher risk of raised ICP [24]. Although SAPVE-VV has a slight advantage in ICV increase, both spring techniques may be considered in syndromic patients. For bicoronal synostosis, SAPVE-C is preferred since it addresses the brachycephalic head shape very well [7], with an optional FOR, as a secondary surgery, if aesthetic concerns persist. Conversely, patients with scaphocephaly or oxycephaly often benefit from SAPVE-VV, which offers simultaneous widening, shortening, and vertical elevation, as well as potential decompression of the posterior fossa when needed.

In non-syndromic patients aged 2 to 4 years, the risk of secondary raised intracranial pressure (ICP) following surgery is generally low, though not negligible. If the patient presents with additional risk factors for elevated ICP—such as multisuture craniosynostosis, a history of previous cranial surgeries, or the presence of a VP shunt—SAPVE-VV is the preferred surgical approach due to its capacity to achieve greater ICV expansion. In contrast, for patients without these risk factors, including those with single-suture craniosynostosis, PVE-S is typically sufficient.

PVE-S is typically selected for patients older than 4 years [7, 9, 25], as the majority of brain growth has occurred by this age, reducing the need for extensive additional space [26]. Interestingly, our data showed a mild trend toward increased ICV gain with advancing age in the PVE-S group, suggesting it may be better suited to older patients. Nonetheless, SAPVE-VV achieved larger volume increases in this age group overall and should be considered when ICP management is the primary goal.

This study has limitations that should be acknowledged. First, its retrospective design and lack of randomized treatment allocation introduce potential selection bias, as surgical techniques were chosen based on patient age, syndromic status, and cranial morphology, rather than by controlled assignment. As a result, the cohorts are not entirely comparable, and differences in outcomes may partly reflect underlying patient characteristics rather than the surgical method alone. Second, although follow-up exceeded 12 months in all cases, longer-term outcomes, particularly the risk of late raised intracranial pressure, may not be fully captured. Finally, this is a single-center experience, and while the uniform surgical protocols and multidisciplinary decision-making strengthen the internal validity, external validation across different centers and surgical teams will be necessary to confirm the applicability of our proposed framework.

## Conclusion

 In summary, posterior vault expansion can be achieved with static remodeling, spring-assisted classic, and spring-assisted vertical vector techniques, each offering distinct advantages depending on patient characteristics. Our findings suggest that SAPVE-C is most effective in younger, often syndromic infants where rapid and minimally disruptive expansion is needed, while SAPVE-VV provides consistent volumetric gains across age groups and may be particularly useful in complex cranial morphologies. PVE-S, although associated with smaller overall volumetric increases, remains a reliable option in older non-syndromic patients. Rather than indicating superiority of one method over another, these results highlight the importance of tailoring the surgical approach to the patient’s age, diagnosis, and cranial morphology. The proposed framework is intended as a practical guide based on our institutional experience, but future prospective and multi-center studies will be required to validate and refine this approach.

## Supplementary Information

Below is the link to the electronic supplementary material.

ESM 1 (JPG 184 KB)

ESM 2 (JPG 240 KB)

ESM 3 (JPG 201 KB)

ESM 4 (MP4 14.2 MB)

ESM 5 (JPG 197 KB)

ESM 6 (MP4 11.3 MB)

## Data Availability

No datasets were generated or analyzed during the current study.
