# Supplementary figures and images for: Surgical management of raised ICP in craniosynostosis: experience-based selection of posterior vault expansion techniques
Source: Childs Nerv Syst. 2025 Oct 2;41(1):303. doi: 10.1007/s00381-025-06961-8 (PMC12491112; doi:10.1007/s00381-025-06961-8)

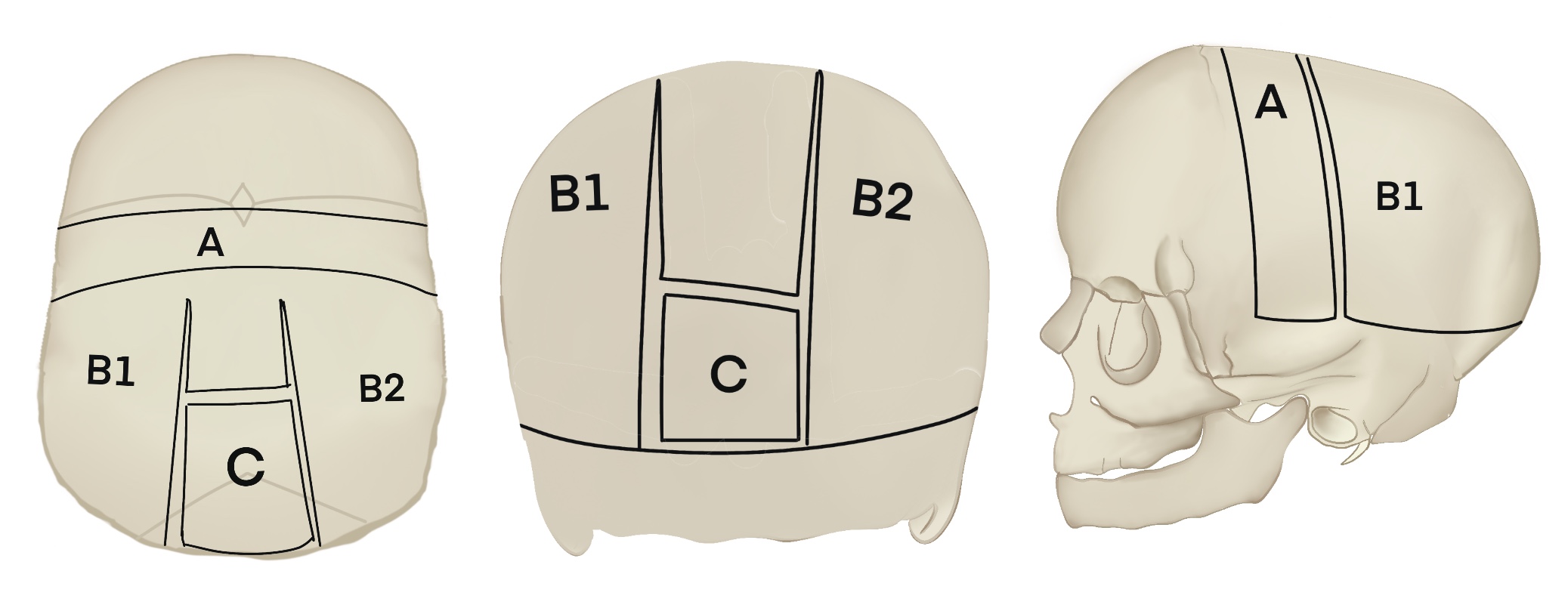

Supplement: Supplementary file 1 — (JPG 184 KB) [file 381_2025_6961_MOESM1_ESM.jpg]

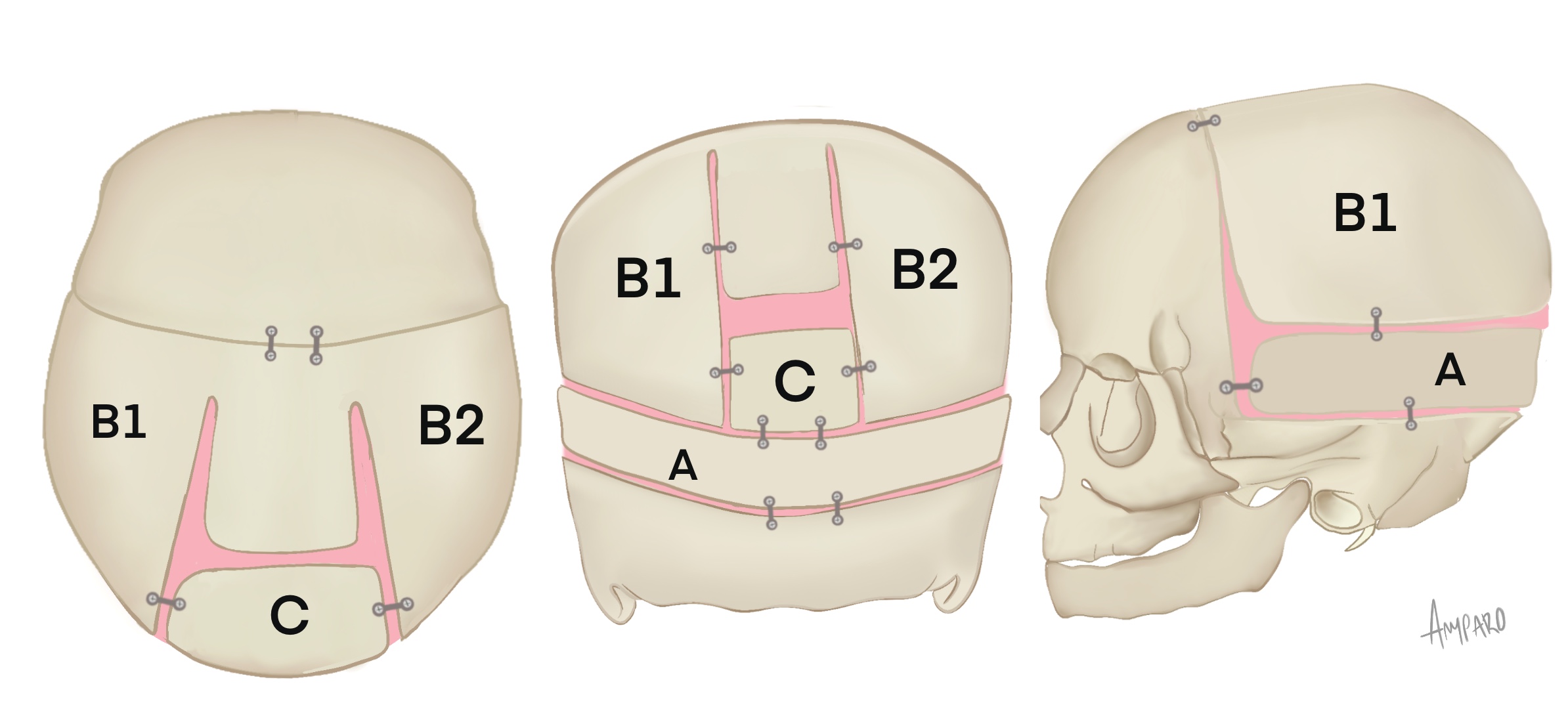

Supplement: Supplementary file 2 — (JPG 240 KB) [file 381_2025_6961_MOESM2_ESM.jpg]

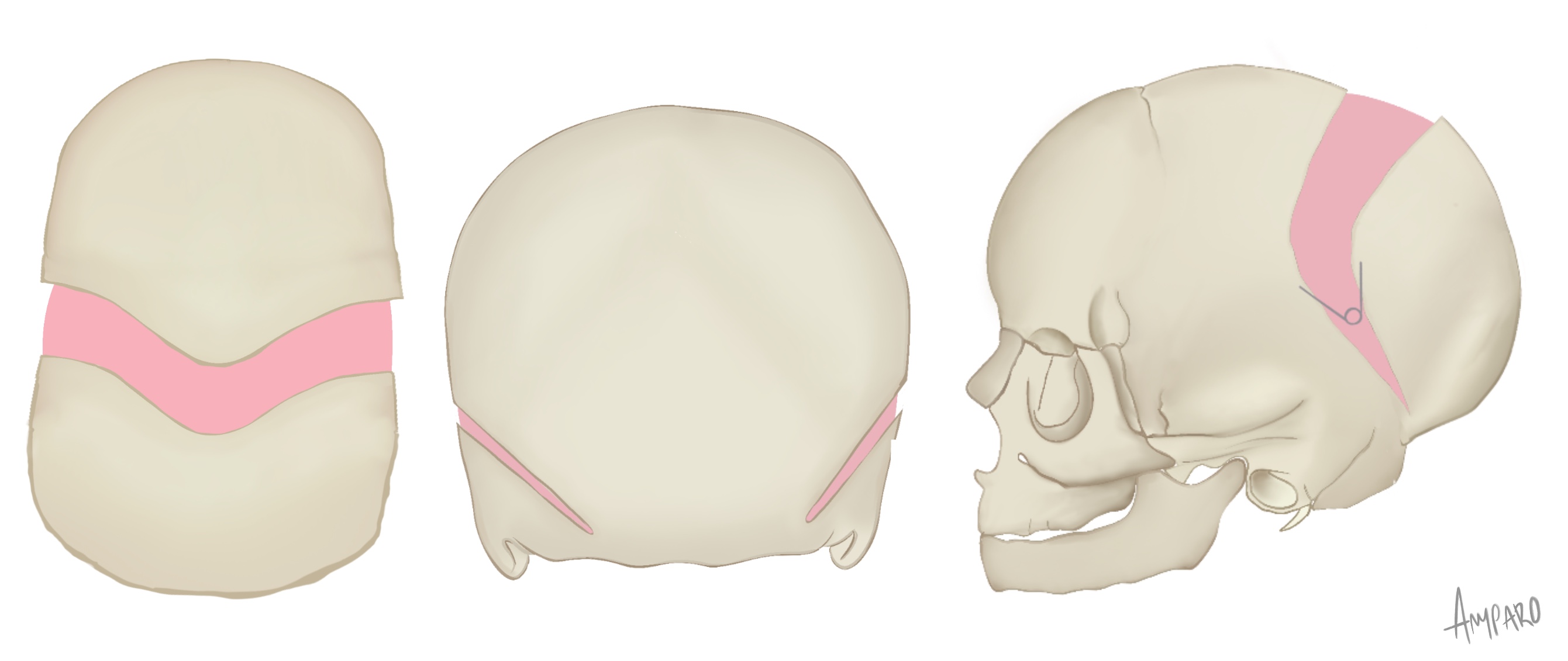

Supplement: Supplementary file 3 — (JPG 201 KB) [file 381_2025_6961_MOESM3_ESM.jpg]

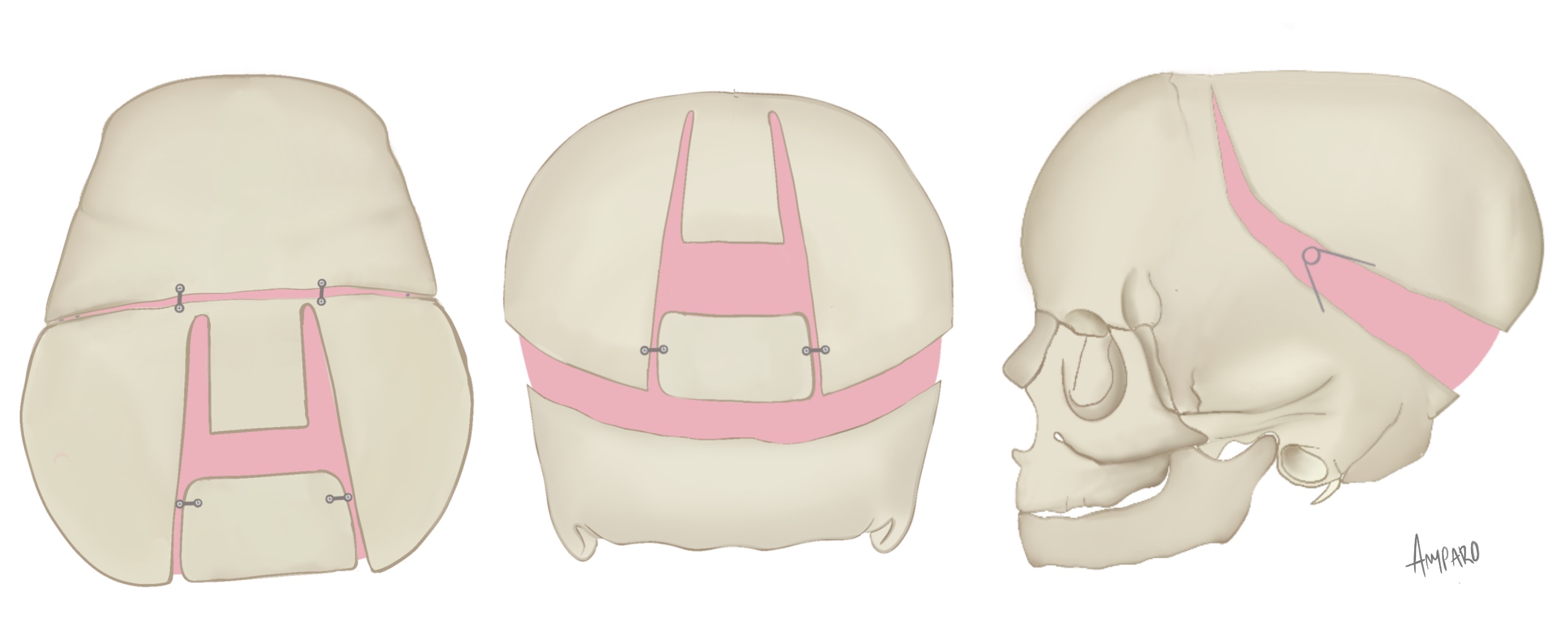

Supplement: Supplementary file 5 — (JPG 197 KB) [file 381_2025_6961_MOESM5_ESM.jpg]
